# Extracting Heart Rate and Temperature Data From Commercial Available Sleep Tracking Devices: Proof of Concept Pilot

**DOI:** 10.1093/geroni/igaf122.2062

**Published:** 2025-12-31

**Authors:** Ellen McCreedy, Ji Won Chung, Corinne Roma, Ann Reddy, Melissa Simonian

**Affiliations:** Brown University, School of Public Health, Providence, Rhode Island, United States; Brown University, Providence, Rhode Island, United States; Brown University, Providence, Rhode Island, United States; Brown University, Providence, Rhode Island, United States; Program of All-Inclusive Care for the Elderly, Riverside, Rhode Island, United States

## Abstract

One of the barriers to using commercially available sleep tracking devices is that each company maintains proprietary sleep algorithms and individual users are unable to download the data that feed those algorithms. Minute-by-minute heart rate and temperature data are important raw data to sleep researchers, as they may be used to help identify people with potential circadian rhythm disruptions and evaluate the effect of promising interventions. In an ongoing pilot, we are using a man-in-the-middle (MiTM) software program to extract user data in real-time from two commercially available sleep tracking devices – a biometric wearable (smartwatch) and a sleep tracking mat. To date, the software has been tested in four community dwelling adults with dementia, each of whom used both devices for three consecutive nights. Caregivers also completed a nightly sleep diary. For the smartwatch, we were able to extract minute-by-minute heart rate and temperature data for three of the four participants. However, despite promising lab tests, the sleep tracking mat failed to connect to our MiTM software in the field. Median differences between caregiver- and device-reported sleep and wake times were similar for both devices. Both devices were feasible and acceptable to all four participants and their caregivers, with a slight caregiver preference for the mat. While data collection is ongoing, we hope to share our MiTM methods and some early lessons learned. Our goal is to increase the transportability and generalizability of research findings across sleep tracking devices, especially given frequent changes in device availability in the marketplace.

